# Effects of Porosity on Piezoelectric Characteristics of Polyvinylidene Fluoride Films for Biomedical Applications

**DOI:** 10.34133/bmef.0009

**Published:** 2023-07-07

**Authors:** Jack T. Kloster, Matthew J. Danley, Victor K. Lai, Ping Zhao

**Affiliations:** ^1^ Advanced Materials Science, University of Minnesota-Duluth, Duluth, MN 55812, USA.; ^2^ Department of Chemical Engineering, University of Minnesota-Duluth, Duluth, MN 55812, USA.; ^3^ Department of Mechanical and Industrial Engineering, University of Minnesota-Duluth, Duluth, MN 55812, USA.

## Abstract

*Objective*: The objective of this work is to study the effects of porosity on mechanical and piezoelectric properties of polyvinylidene fluoride (PVDF) films for biomedical applications. *Impact Statement:* By investigating the piezoelectric properties of PVDF and the porosity effect on its electromechanical performance, there is potential for further development of PVDF as a hemodynamic sensor that can lead to further technological advancements in the biomedical field, benefiting patients and physicians alike. *Introduction*: PVDF thin films have shown potential in the application of hemodynamic flow sensing and monitoring the effects on blood flow caused by prosthetic valve implantation via the transcatheter aortic valve replacement operation. The piezoelectric performance of PVDF films can be influenced by the porosity of the material. *Methods*: In this study, strain tracking was performed on thin film PVDF specimens with various levels of porosity and pore sizes to determine the mechanical properties of the specimens. The mechanical properties were used to model the PVDF material in COMSOL multiphysics software, in which compression test simulations were performed to determine the piezoelectric coefficient *d*_33_ of the PVDF. *Results*: A decline in the elastic modulus was found to be highly inversely correlated with porosity of the specimens and the simulation results show that elastic modulus had a much greater effect on the piezoelectric properties than Poisson’s ratio. *Conclusion*: A combination of experimental and computational techniques was able to characterize and correlate the mechanical properties of PVDF films of varying porosities to their piezoelectric properties.

## Introduction

Piezoelectric materials are a unique branch of materials that can generate electric potentials when subjected to mechanical stress. This ability makes piezoelectric materials uniquely suited for use in pressure sensory and related applications. Polyvinylidene fluoride (PVDF) is a piezoelectric polymer material that has shown great aptitude as a pressure and flow sensor, especially in biomedical applications [1–5]. Sharma et al. [6] have demonstrated in multiple publications that PVDF as a thin film can be used as a pressure sensor integrated with a catheter for measuring intravascular forces during surgery. In a 2003 study, a PVDF thin film material was developed for monitoring cardiorespiratory conditions during sleep for patients with cardiorespiratory sleep disorders [7]. The sensor device could be placed under the patient and measure the forces caused by heartbeats and diaphragm movement during sleep to monitor health. This technology was further developed by Choi et al. in 2006 when they integrated the PVDF film sensor in a wearable belt device [8].

In applications as a flow sensor, PVDF has been developed for measuring flow rates and pressures of both liquids and gases alike, proving that it is a material capable of consistently generating electric signals proportional to the surrounding pressure [9], while remaining robust, compact, and relatively simple to produce [10]. PVDF also has applications in the biomedical industry as a flow sensor for measuring the flow rate of blood in the portal vein during liver transplant surgery [11]. Patients who suffer from liver cirrhosis often develop portosystemic shunts that divert blood flow away from the portal vein due to hypertension in the liver. Upon receiving a new liver, this reduced blood flow may lead to continued liver damage. Alternatively, if the patient receives a partial liver, an overflow of blood can similarly cause damage. Therefore, it is crucial to monitor and control the flow of blood through the portal vein in patients undergoing liver transplant surgery. Previously, portal vein blood flow was measured using ultrasound technology, which is a method highly dependent on operator expertise and experience. Another monitoring method used involved a thermal sensor located on the end of a catheter. Cold saline solution was mixed with the blood, and the temperature sensor could determine the flow rate based on the rate of temperature change and the concentration of saline solution in the patient’s blood. However, this method made continuous monitoring impractical since the concentration of saline in the blood was unsustainable. As an alternative solution for monitoring blood flow in the portal vein, a spirally wound PVDF thin film was developed to be implanted in the vein. The PVDF sensor was determined to effectively measure flow rate with a sensitivity of 0.02 mV peak-to-peak per 20 ml/min over a range of flow rates from 600 to 1,800 ml/min. The sensor’s measurements were also determined to be stable over a duration of 120 min [11].

By enhancing the piezoelectric characteristics of PVDF, the material can be improved to be a more sensitive, consistent, and robust sensor, suited for a wide variety of applications. The PVDF polymer structure is primarily made up of 3 different copolymers, referred to as α-, β-, and γ-phases. The α-phase of PVDF is the most stable of the conformations and typically the most dominant in the polymer structure immediately after synthesis. However, the α-phase does not exhibit a dipole, and thus does not contribute to the piezoelectric characteristics of the material [12]. The β-phase exhibits the greatest dipole of the conformations and is thus most associated with the piezoelectric properties of PVDF. The γ-phase is similarly polar. However, it typically makes up a much smaller portion of the overall structure, giving it a smaller influence on the material’s properties. There exist a few common methods used for increasing the concentration of β-phase within PVDF. Mechanical stretching on the material can result in increased alignment of the amorphous regions in the polymer and can also lead to transformation from α-phase to β-phase, improving the piezoelectric characteristics. Electric poling is another method for improving β-phase concentrations. By applying high voltages across the material, nonpolar conformations can be transformed into the polar β-phase. Additionally, both the stretching and poling methods can benefit from being performed in conjunction with thermal annealing, which acts to increase the conformations’ susceptibility to transformation [12].

β-phase formation in PVDF has also been shown to be influenced by conditions during its synthesis. Chinaglia et al. [13] showed that the solvent evaporation rate during solution casting of PVDF can greatly influence the presence of β-phase within the polymer, with PVDF solutions that were cast at lower temperatures found to exhibit slower solvent evaporation rates and significantly larger concentrations of β-phase. In a 2008 study [14], various solvents were used for solution casting of PVDF, and the effects of their evaporation rates on the formation of β-phase were studied. It was determined that solvents with higher boiling points exhibited slower evaporation rates, and thus led to the formation of more β-phase under identical temperature conditions. Therefore, solvent evaporation rate is a crucial factor to control during synthesis of PVDF via solution casting since it can greatly affect the piezoelectric properties of the material.

In addition, porosity has become a factor of great interest with respect to the piezoelectric performance of PVDF. Increasing the number and size of pores within the material can increase the localized micromechanical strain under identical loading conditions. This increased strain leads to larger charge separations at the molecular level, resulting in greater electric polarization and improved overall piezoelectric performance [15]. There are multiple methods for introducing porosity in PVDF films during synthesis. One method is by mixing solvents. The mixed-solvents method is founded on the principle that 2 mixed solvents will have different evaporation rates. As one solvent evaporates more rapidly, the surface tension will begin to change, and water droplets will form on the surface of the PVDF film. As these water droplets evaporate, pores in the surface of the material will be left in their place. By mixing acetone and 2-butanone, this method of introducing pores in PVDF has been shown to improve piezoelectric performance by as much as 107% [15]. Another method for introducing void regions in PVDF is by integrating pore-inducing particles in the polymer solution during the solution casting process [16]. A 2014 study [17] utilized this method for introducing pores into PVDF thin films for energy harvesting under vibrational loads. During the synthesis process, zinc-oxide (ZnO) nanoparticles were added to the PVDF solution before solution casting. Once the films were cast, they were bathed in hydrochloric acid (HCl), which dissolved the ZnO particles, leaving behind nano-scale pores in their place. ZnO particles were selected to create the nano-pores because they are low-cost, non-toxic, scalable, and readily removable by acid compared with other inorganic particles such as SiO_2_ [18–20]. In this study, PVDF specimens were synthesized with various ZnO mass fractions ranging from 10% to 70% ZnO by mass before etching. The greatest piezoelectric performance was exhibited at a removed ZnO mass fraction of 50%. At higher mass fractions of ZnO, the voltage signals generated by the PVDF films began to decrease, likely due to the overall reduction of PVDF material in the films [17]. From these results, the piezoelectric performance of PVDF thin films can greatly benefit from the introduction of pores within the material’s structure. It is envisioned that the PVDF films modified by the nanoporous structures are well suitable for biosensing applications such as monitoring hemodynamic flow during in vitro studies of blood flow in the aorta after implantation of a prosthetic valve via the transcatheter aortic valve replacement (TAVR) due to their high piezoelectric coefficients, flexibility, and low weight.

The goal of this study is to further investigate the piezoelectric performance of PVDF thin films, specifically how performance is affected by the introduction of porosity via ZnO etching under compression, which is applicable as a biosensor for hemodynamic pressure sensing. PVDF specimens, synthesized using the solution casting method, are tested under compressive stresses to determine their piezoelectric coefficient, *d*_33_. Various mass fractions and varying sizes of ZnO nanoparticles are used to induce a variety of pore volumes and sizes. Furthermore, visual strain tracking software is utilized to determine mechanical properties of the PVDF, and these mechanical properties are used to model a simulated PVDF material via finite element analysis (FEA) simulation. These simulated results of the piezoelectric properties serve to compare and validate the experimental results. By investigating the piezoelectric properties of PVDF and the porosity effect on its electromechanical performance, there is potential for further development of PVDF as a hemodynamic sensor that can lead to further technological advancements in the biomedical field, benefiting patients and physicians alike.

## Results

### Mechanical testing and strain tracking

The strain data collected via the strain tracking process, along with the load data collected by LabVIEW during tensile tests, were used to plot stress–strain curves for the PVDF samples. Figure S1 shows examples of stress–strain curves for specimens fabricated with various mass fractions of ZnO (only one per wt% ZnO is shown for clarity). As the mass fraction of ZnO increased (meaning increased porosity), the slope of the stress–strain curve decreased, and the material began to yield at lower stresses. This implies a lower elastic modulus, as well as a lower ultimate tensile strength of the material.

**Fig. 1.**
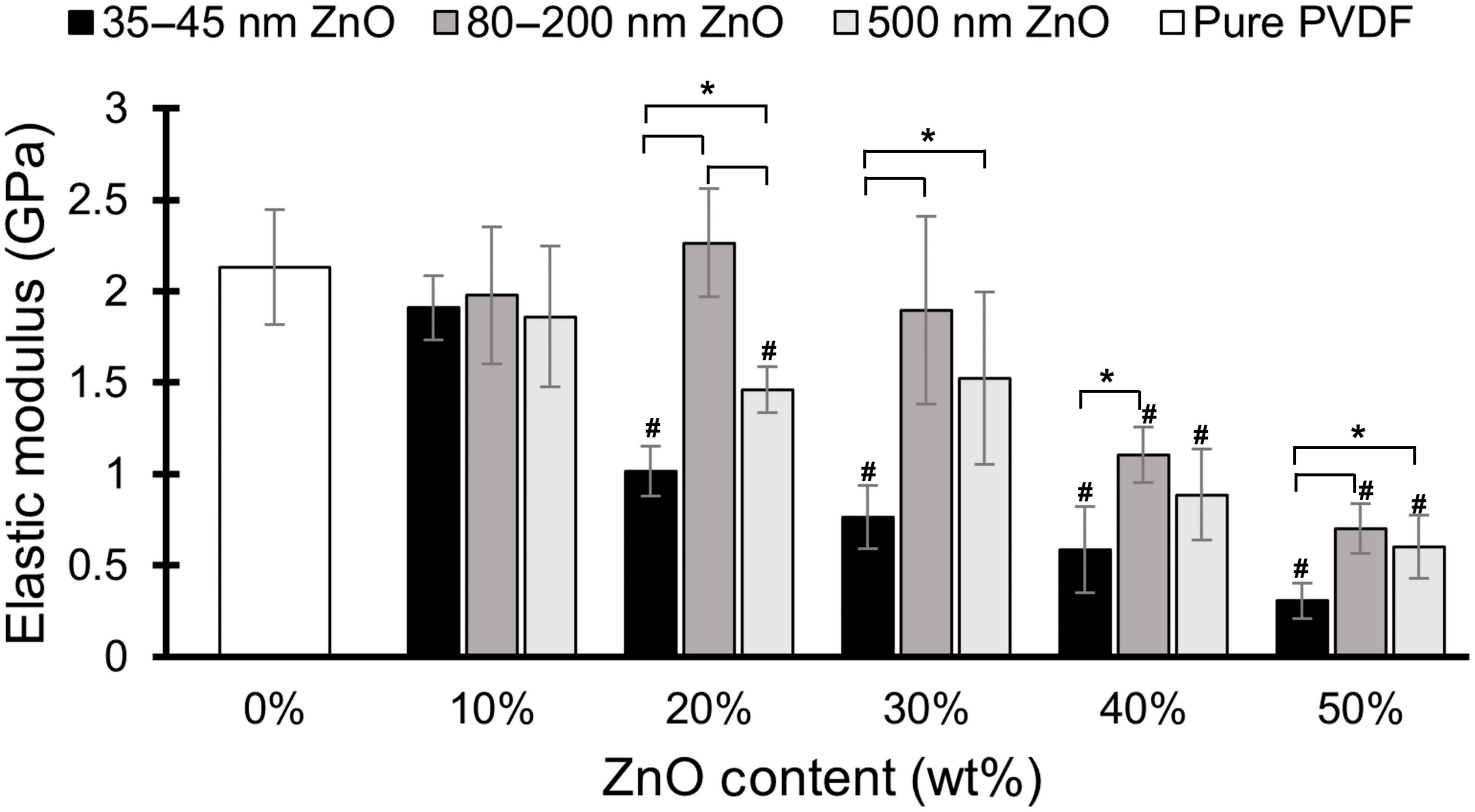
Mean elastic moduli for PVDF specimens with varying ZnO content. The elastic modulus of the PVDF films decreased with increasing ZnO content, regardless of the particle size (*n* = 5, error bars represent ± one standard deviation; # represents statistical difference at *p* < 0.05 compared with the 0 wt% ZnO control samples). In general, an increase in particle size from 35 to 45 nm to larger particles resulted in an increase in elastic modulus across most ZnO content, except for the 10 wt% ZnO, which showed no statistical significance in modulus across all particle sizes (* represents statistical significance at *p* < 0.05).

Figure 1 shows the mean elastic modulus for each group of PVDF specimens. Each column represents the mean elastic modulus of 5 identically fabricated specimens, according to their ZnO content and ZnO particle size. Elastic moduli were determined by measuring the slopes of the stress–strain curves in the elastic regions. As the mass fraction of ZnO increased, the elastic modulus of the PVDF was found to decrease. This result confirms expectations that samples with a greater porosity will exhibit more strain under identical stress conditions, which implies that a larger charge separation may be occurring as well. The mean elastic modulus of the pure PVDF specimens was found to be 2.13 GPa. The smallest elastic moduli for each size of ZnO occurred at a mass fraction of 50% ZnO, with moduli values of 0.31 GPa, 0.70 GPa, and 0.60 GPa for the 35- to 45-nm, 80- to 200-nm, and 500-nm ZnO specimens, respectively. These represent a reduction in elastic modulus by 86%, 67%, and 72%, respectively, compared to the pure specimens. The PVDF samples made with 35- to 45-nm ZnO particles exhibited the most rapid falloff in elastic modulus as ZnO content increased, dropping significantly with just 20% ZnO content. The specimens made with 80- to 200-nm ZnO particles did not see significant drops in elastic moduli until ZnO content reached 40%, at which point the results fell sharply. The 500-nm ZnO samples showed a more gradual decline in elastic modulus as ZnO content increased, with a noticeable decrease by 20% ZnO.

**Fig. 2.**
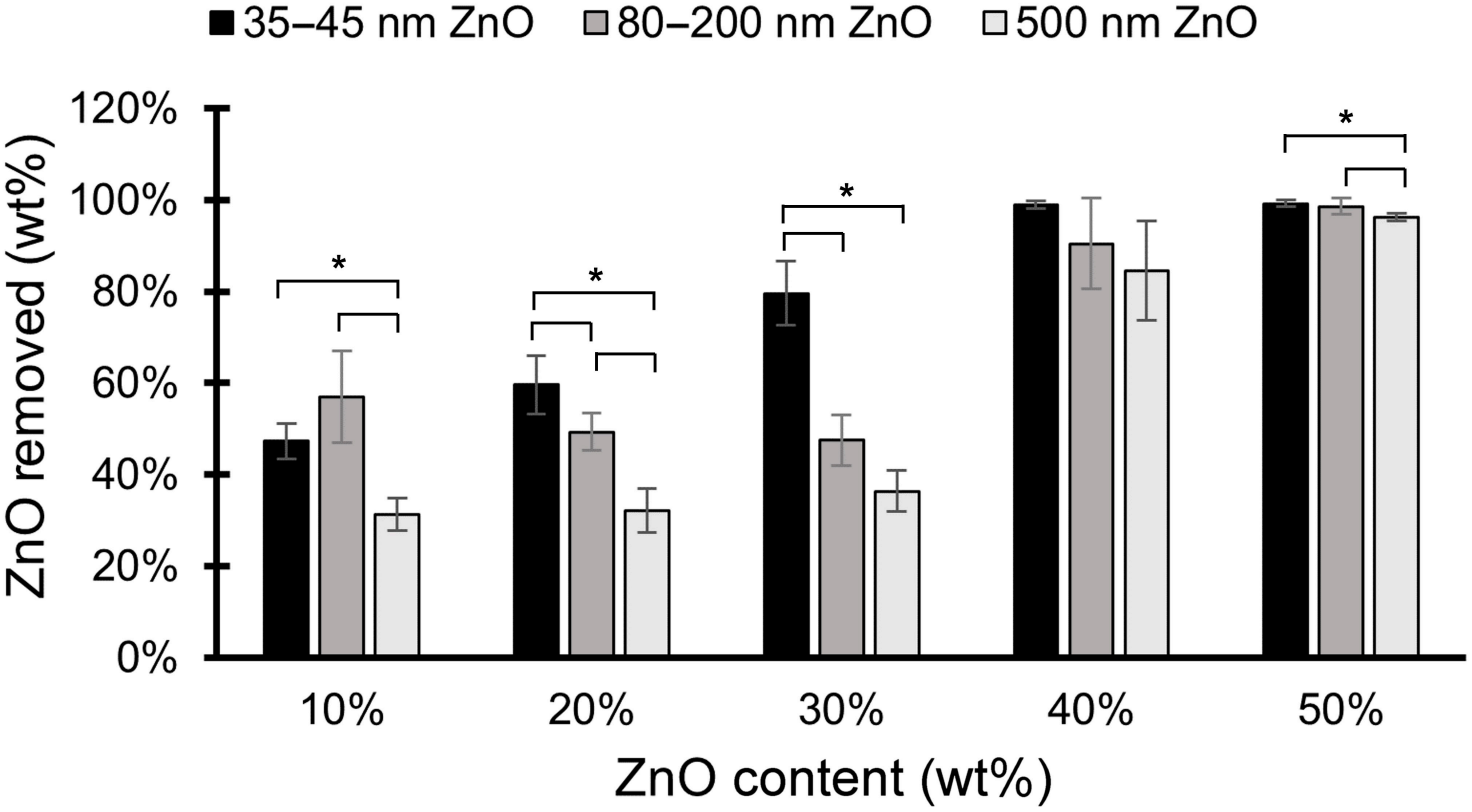
Mean fraction of ZnO removed for PVDF samples of various ZnO content. In general, an increasing amount of ZnO was removed with increasing initial ZnO content (*n* = 4; error bars represent ± one standard deviation). In general, larger particle sizes induced a larger fraction of ZnO removed from the samples across all ZnO contents, although some differences were not statistically significant (* represents statistical significance at *p* < 0.05).

Figure 2 shows the weight percent of ZnO removed from the PVDF specimen versus the weight percent of ZnO content added to the specimen. The data show that in specimens made with 10% to 20% ZnO content, less than 60% of the ZnO was removed during the 24-h HCl bath for all particle sizes. In samples with 40% to 50% ZnO content, however, nearly 100% of ZnO mass was removed for all samples. This disparity in ZnO removal is likely due to ZnO particles being trapped in the PVDF. At lower concentrations of ZnO, a greater amount of ZnO particles will be enveloped by the surrounding PVDF, restricting interaction between the ZnO particles and the HCl as the porous channels are presumably less interconnected. At higher concentrations of ZnO, more complete pathways form in the material for HCl to diffuse, resulting in a greater fraction of ZnO removed.

In addition, the 35- to 45-nm ZnO particles were removed in greater fractions over the 20%, 30%, and 40% ZnO content samples, while the 500-nm ZnO particles were consistently the least efficiently removed. This may be due to the smaller particles dissolving more easily in the HCl, or that the smaller particles may distribute more homogeneously throughout the PVDF, creating more interconnected pathways for the HCl. The ZnO removal data are consistent with the elastic modulus data in that the samples with 35- to 45-nm ZnO particles exhibited an immediate increase in ZnO removal and an immediate decrease in elastic modulus. The samples made with the 80- to 200-nm ZnO particles, however, only exhibited significant changes in ZnO removal and elastic modulus when ZnO content reached 40%.

**Fig. 3.**
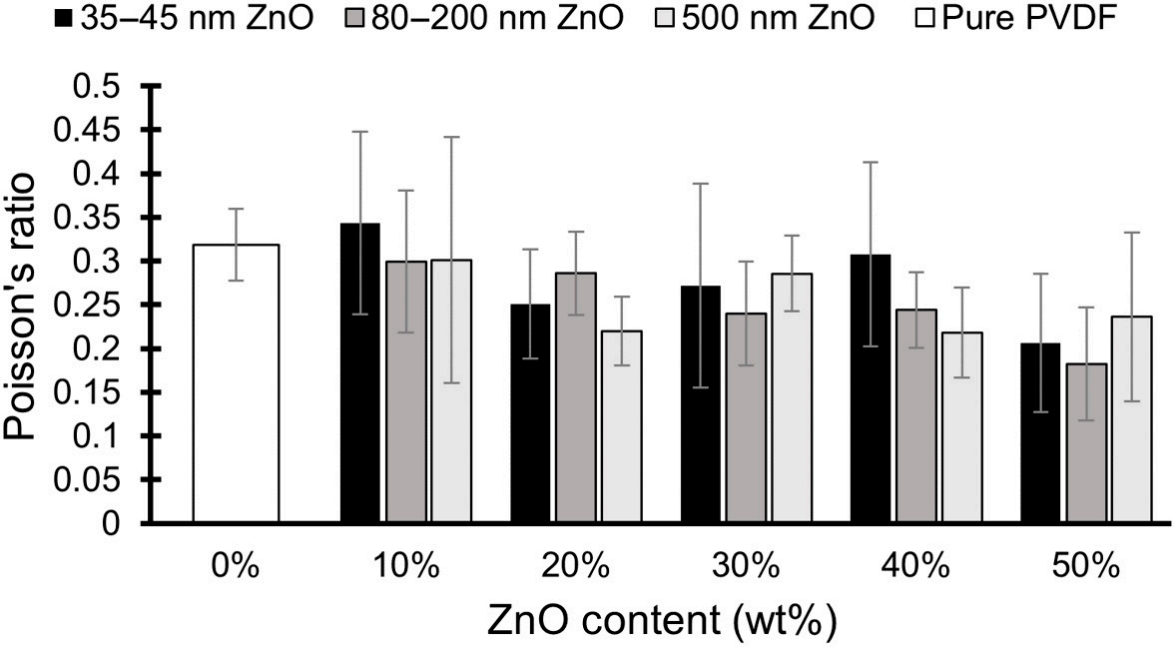
Mean Poisson’s ratios for PVDF specimens with various ZnO content. No statistically significant differences at the 0.05 level were found between ZnO particle size and Poisson’s ratio across all ZnO contents (*n* = 4 to 5 in each case; error bars represent ± one standard deviation).

Figure 3 shows the mean Poisson’s ratio for each group of 5 PVDF samples according to their ZnO content and ZnO particle size. The mean Poisson’s ratio for the pure PVDF specimens was determined to be 0.32, and the ratio was shown to gradually decline as ZnO content was increased. At their lowest, Poisson’s ratios of specimens with 50% ZnO reached an average of 0.20, although large standard deviations within the groups of just 5 samples made it difficult to conclude the true means. There was found to be no statistically significant correlation between ZnO particle size and Poisson’s ratio across all ZnO contents (*p* = 0.10).

### Simulated *d*_33_ results and comparison with experimental data

**Fig. 4.**
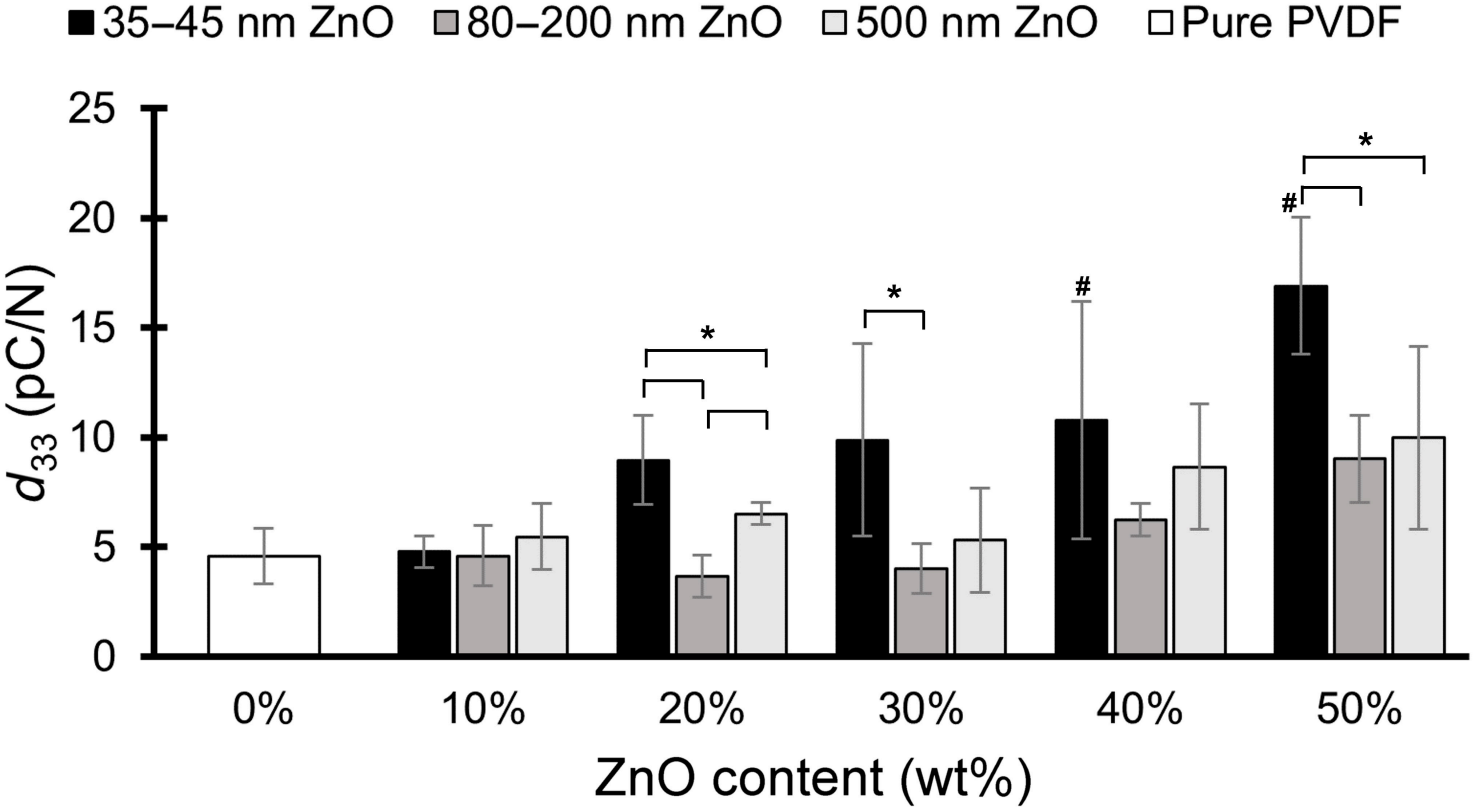
Mean simulated *d*_33_ values for PVDF with various ZnO content. In general, *d*_33_ values showed a general increasing trend with initial ZnO content, with the smallest particle size of 35 to 45 nm exhibiting the highest values (*n* = 4 to 5 from experimental data of elastic modulus and Poisson’s ratio, error bars represent ± one standard deviation; # represents statistical difference at *p* < 0.05 compared with the 0 wt% ZnO control). Across particle sizes, the smallest particle size samples exhibited higher *d*_33_ values compared to the larger size samples, although not all differences were statistically significant (* represents statistical significance at *p* < 0.05).

The elastic modulus and Poisson’s ratio values were used as inputs to the elasticity matrix of the porous PVDF material to edit the PVDF material properties in the COMSOL simulation. The results of the *d*_33_ values, normalized for the total mass of PVDF in the samples, are plotted in Fig. 4. The simulated pure PVDF films were found to have a mean *d*_33_ value of 4.8 pC/N, and the greatest *d*_33_ values in all cases were observed at a ZnO content fraction of 50% (*p* < 0.01). At 50% ZnO content, the samples fabricated with 35- to 45-nm, 80- to 200-nm, and 500-nm ZnO particle sizes exhibited mean *d*_33_ coefficients of 16.9, 9.0, and 10.0 pC/N, respectively. These represent increases relative to pure PVDF of 270%, 96%, and 118%, respectively. PVDF specimens made with 35- to 45-nm ZnO particles exhibited the largest increase in *d*_33_ at all ZnO content levels of 20% and above. This is consistent with the fact that those 35- to 45-nm ZnO samples showed the greatest levels of ZnO removal during etching, as well as the smallest elastic moduli. This is in line with the assumption that PVDF with greater porosity will exhibit a smaller elastic modulus and thus undergo greater strain per stress, leading to larger charge separations and higher piezoelectric performance. The samples with 80- to 200-nm and 500-nm ZnO particles did not see significant increases in *d*_33_ coefficients until ZnO content reached 40% to 50%. Again, this observation correlates with the trend seen in the ZnO removal and elastic modulus findings.

**Fig. 5.**
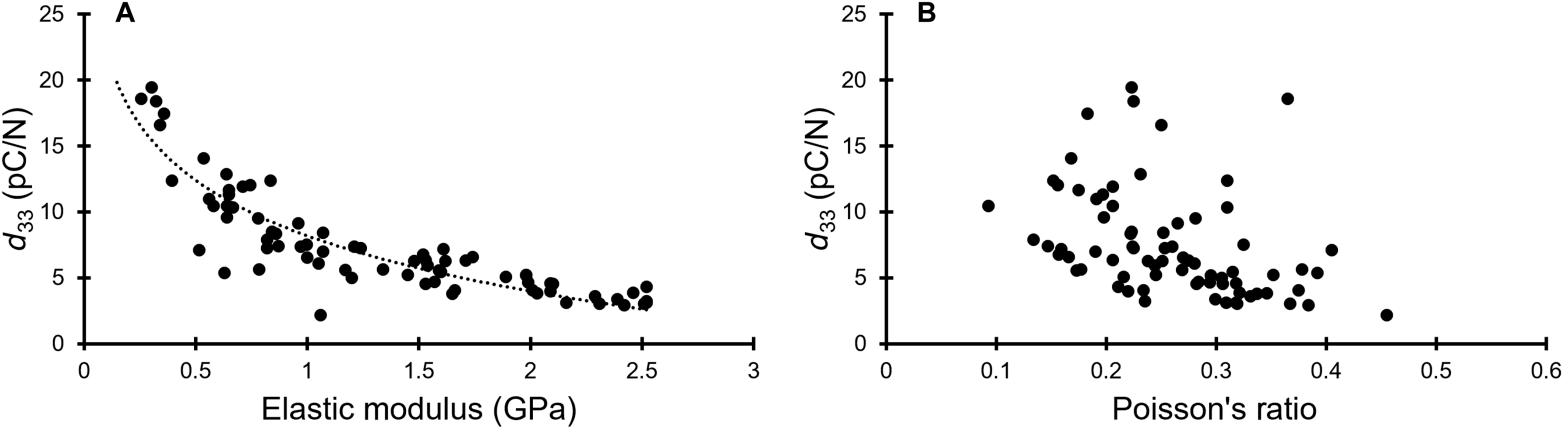
*d*_33_ versus (A) elastic modulus and (B) Poisson’s ratio. The *d*_33_ values decreased exponentially with increasing elastic modulus, but did not change significantly with Poisson’s ratio.

To further investigate the relationship between the piezoelectric coefficients and the material properties of PVDF, the simulated *d*_33_ values were plotted against the elastic modulus value and Poisson’s ratio in Fig. 5A and B, respectively. Figure 5A suggests an exponential decrease in piezoelectric coefficient with increasing elastic modulus. With Poisson’s ratio, however, no discernable or significant relationship was found with the piezoelectric coefficient (Fig. 5B). Taken together, these simulation results suggest that the elastic modulus of the PVDF material has a much greater bearing on the overall piezoelectric performance of the material than Poisson’s ratio.

### Experimental results and comparisons

**Fig. 6.**
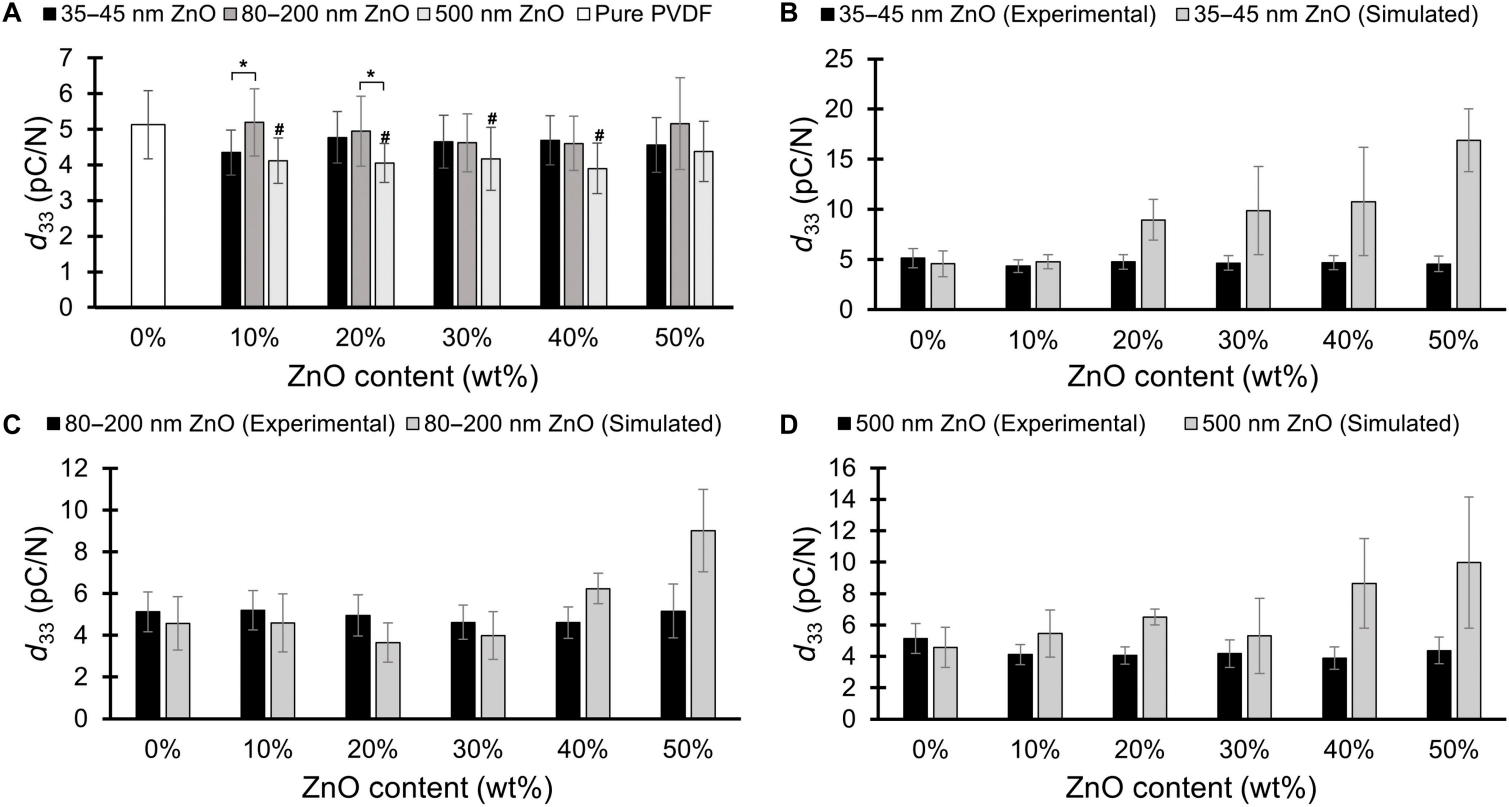
*d*_33_ values obtained from experimental data (A), and comparison of experimental data with simulation results for various particle sizes (B to D). When compared with the experimental *d*_33_ values of the 0 wt% ZnO control samples, only the largest particle size samples for 10 wt% to 40 % ZnO content were significantly different at *p* < 0.05 (denoted with *#* in (A)). In addition, few significant differences in experimental *d*_33_ values were observed between samples of different particle sizes across all wt% ZnO (*p* < 0.05 denoted with * in (A)). These results are not predicted by the simulations, which showed an increasing trend in *d*_33_ with increasing wt% ZnO (B to D) (*n* = 23 to 25 for the experimental *d*_33_ data in (A); error bars represent ± one standard deviation).

Figure 6 shows the *d*_33_ coefficients of the various samples obtained from the compression tests compared with the simulation results. Experimentally (Fig. 6A), the mean *d*_33_ of the pure PVDF samples was 5.1 pC/N; as ZnO content increased, the mean *d*_33_ values ranged between 4 and 5 pC/N without any definitive trends. When excluding the pure PVDF specimens, there was no statistically significant difference in *d*_33_ for samples with varying ZnO content levels (*p* = 0.30). There was, however, a significant difference in *d*_33_ for samples with varying ZnO particle sizes (*p* < 0.01), with films fabricated using 500-nm ZnO particles averaging lowest at approximately 4 pC/N, and the 80- to 200-nm ZnO particle samples averaging the highest in the range of 4.6 to 5.2 pC/N.

Comparison of experimental data with simulation results in Fig. 6B to D shows that the experimental results largely did not match what was predicted from the FEA simulations. For all particle size cases, the experimental *d*_33_ values were relatively similar across all ZnO content. Unlike the simulation results, the experimental data did not exhibit an increase in *d*_33_ values that the simulated results predicted once the ZnO content increased and the elastic modulus decreased.

## Discussion

This study uses a combination of experimental techniques to probe the mechanical and piezoelectric properties of porous PVDF thin films, and compared these results with predictions from computational simulations using COMSOL. A decline in the elastic modulus was found to be highly inversely correlated with the measured fraction of ZnO removed from the specimens, which is an expected result as a more porous material would have a lower stiffness. Interestingly, the samples synthesized with 500-nm ZnO particles exhibited a smaller elastic modulus than those with 80- to 200-nm ZnO particles, despite having less mass of ZnO removed. This may be because of the effect of larger size pores on the elastic modulus of the PVDF: Cabanillas-Casas et al. [21] found that the elastic modulus of graphene membrane decreased as nano-scale pores increased in size. Therefore, although the number of pores may be fewer, the elastic modulus may be lower in 500-nm ZnO samples due to the increased pore size.

The simulation results show that elastic modulus had a much greater effect on the piezoelectric properties than Poisson’s ratio. In the more porous PVDF films that had lower elastic modulus, the simulated piezoelectric coefficients *d*_33_ were found to be significantly greater, increasing by as much as 270%, when compared to PVDF with no introduced pores. The experimental data, however, did not match this predicted result. Some possible confounding factors for this discrepancy include the following:

1. The concentration of β-phase in the PVDF may be low due to the lack of polarity enhancement techniques used in this study. While the *d*_33_ values for pure PVDF (i.e., 0 wt% ZnO) matched well with the simulated *d*_33_ value, any predicted increase in *d*_33_ due to an increase in porosity could be offset by a decrease in the relative amounts of the electropositive β-phase. Further analysis of the phase composition in these films would have to be performed to test this hypothesis.

2. The piezoelectric performance of the films is likely affected by the ZnO particles that remain unetched in the samples. Any remaining ZnO particles trapped within the PVDF film could induce changes to its overall mechanical properties and composition of these films, all of which could affect the piezoelectric properties in ways that may not be easily decoupled. Even in the 50 wt% ZnO samples where the bulk of the ZnO was removed, the experimental *d*_33_ was significantly smaller than predicted values from our simulations. This interesting result suggests that the dissolution process may have induced profound changes to the microstructure of these films. It is unclear whether the pores remain intact upon dissolution of the ZnO particles (i.e., the porous structure did not collapse on itself). Visual confirmation of the porous structure (e.g., using scanning electron microscopy) would be helpful for semi-quantitative analysis of the final microstructure within these films.

3. The β-phase concentration in the PVDF films may have been low due to a high solvent evaporation rate during solution casting. Li et al. [9] suggest that the nonpolar α-phase of PVDF begins to form during solution casting when solvent evaporation rates rise above 4.1 mg/min. For the first 90 min in the oven, mean evaporation rates ranged from 40 to 50 mg/min for the first 90 min and 80 to 90 mg/min thereafter, before falling off rapidly as the majority of 2-butanone evaporated (see Fig. S2). These evaporation rates, which are far greater than those suggested in literature for forming β-phase, may be significantly contributing to a lack of β-phase formation, and thereby hindering the piezoelectric performance of the PVDF films.

In summary, understanding how material properties such as the elastic modulus and Poisson’s ratio affect the piezoelectric performance of PVDF films, and how those properties can be controlled by altering the porosity of the material, is crucial for the further development of PVDF as a pressure-sensing material. By improving the piezoelectric performance of PVDF, more sensitive and reliable pressure sensors can be developed for biomedical applications like hemodynamic monitoring. One potential application is the development of repeatable bench-top tests for studying how a TAVR implant affects blood flow, as well as how the artificial valve interacts with the structure of the heart, to better understand certain complications that arise from a TAVR procedure. Such a bench-top setup would require flexible sensors placed within the artificial aortic arch segment to accurately track changes in blood pressure and/or flow rate before and after implantation; thin porous PVDF films have the potential to support such technological advancements that will improve patient outcomes.

## Materials and Methods

### Experimental and technical design

**Fig. 7.**
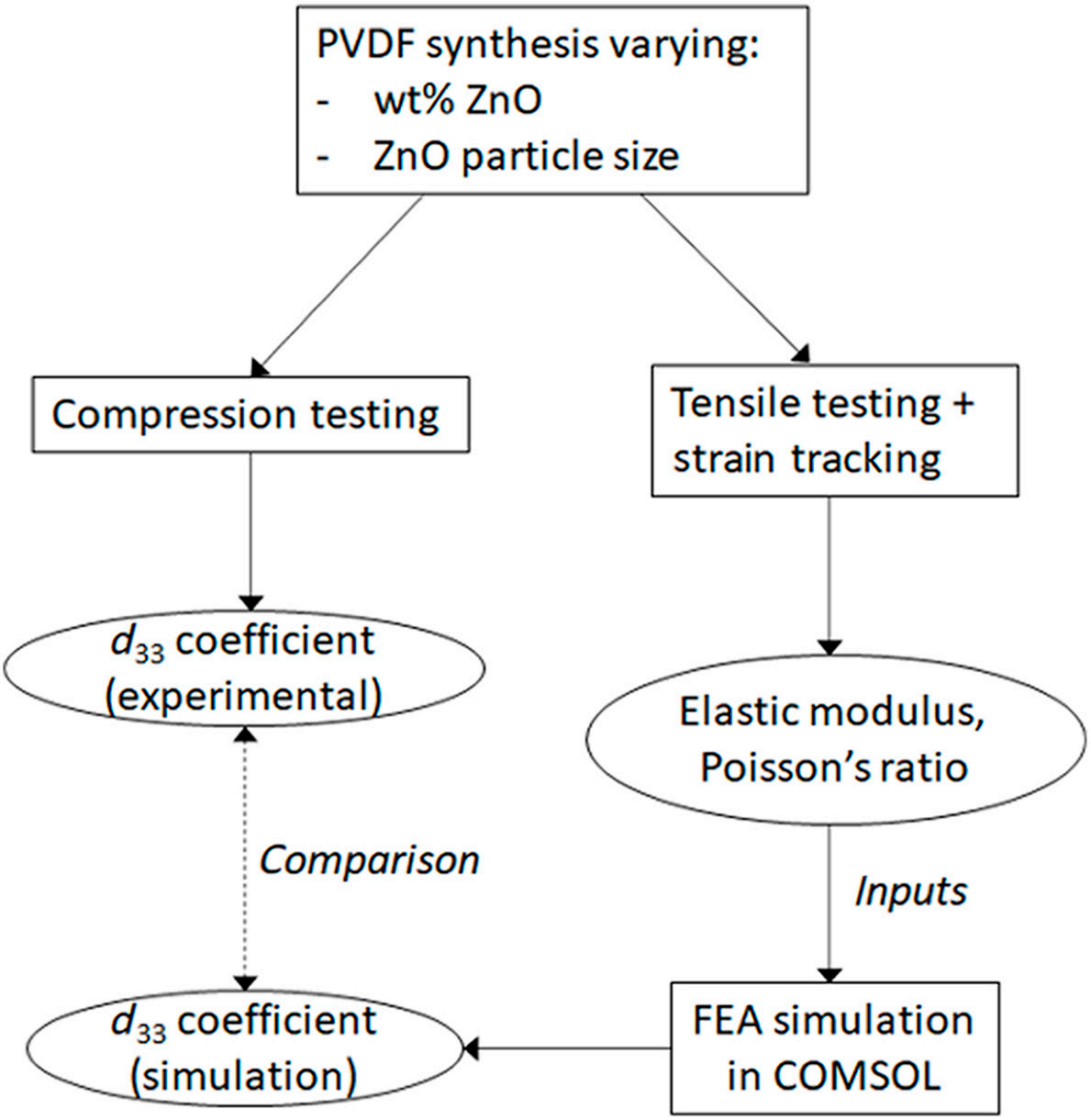
Flowchart detailing the experimental and computational design in this study.

The objective of this study is to characterize and correlate the mechanical properties of PVDF films of varying porosities to their piezoelectric properties using a combination of experimental and computational approaches. Tensile tests coupled with strain tracking of the films during stretching were performed to obtain the elastic moduli and Poisson’s ratios of the samples; these properties are then used as input parameters into the computational model using COMSOL Multiphysics software. Subsequently, the samples were subjected to compression tests and the electrical voltage generated was collected to estimate the *d*_33_ coefficient of each sample. A flowchart of the entire experimental and computational design is shown in Fig. 7.

### PVDF synthesis and preparation

PVDF samples were synthesized using the solvent casting method. PVDF powder (Sigma Aldrich, St Louis, MO) was placed in a vial and dissolved in 15 ml of 2-butanone (Sigma Aldrich, St Louis, MO). To expedite the dissolution of PVDF, the solution was placed in an oven at 80 °C. The vial was intermittently (approximately once per minute) removed from the oven and shaken by hand to prevent the precipitation of solid PVDF at the bottom of the vial. After approximately 15 min in the oven, the PVDF solution turned from opaque to transparent, indicating that the PVDF had fully dissolved in the 2-butanone, at which point the solution was removed from the oven and the ZnO particles (US Research Nanomaterials Inc., Houston, TX) were added to the solution. In the case of pure PVDF specimens, the solution was poured directly into Petri dishes (without the addition of any ZnO particles), which were placed back in the oven at 80 °C for solvent casting. PVDF-ZnO specimens had ZnO added to the solution to meet the desired mass fraction. Specimens were made with a mix of PVDF-ZnO that ranged from 0% to 50% ZnO by mass, in increments of 10%. The sum of the masses of PVDF and ZnO in the solution was kept at 1 g for all specimens. Three unique diameters of spherical ZnO nanoparticles were used: 35 to 45 nm, 80 to 200 nm, and 500 nm. Ten replicates of each specimen type were made: 5 for tensile testing and strain tracking tests to determine mechanical properties of the PVDF films, and the other 5 for compression tests to determine the *d*_33_ piezoelectric coefficient.

Once the ZnO particles were added to the solution, the solution was mixed using a vortex mixer and subsequently placed in an ultrasonication bath to break up the ZnO particles and facilitate dissolution. Samples remained in the ultrasonication bath, being removed and vortexed occasionally, until ZnO particles no longer accumulated at the bottom of the vial, which indicated complete dissolution. Once the ZnO was dissolved, the solution was poured into Petri dishes for solvent casting. The Petri dishes were preheated in the oven at 80 °C to mitigate the amount of 2-butanone condensation on the inside of the Petri dish cover. Samples were heated in the oven at 80 °C until the 2-butanone was fully evaporated, leaving a PVDF-ZnO thin film approximately 70 μm in thickness. To dissolve the ZnO particles and thus introduce porosity into the thin films, the PVDF-ZnO specimens were bathed in 10 ml of HCl (Sigma Aldrich) for 24 h while continuously stirring with a magnetic stir bar. The specimens were then removed from HCl and soaked briefly in deionized water to wash off the remaining HCl. To estimate the percent of ZnO removed from the samples, specimens were weighed prior to the HCl bath, and weighed again after a total of 24 h in HCl. Once the samples were dry, they were then ready to be prepared for testing.

### Tensile testing and strain tracking

For tensile testing and strain tracking, 5 cm × 2 cm rectangular specimens were cut from the specimens and finely speckled with black spray paint so that the speckles could act as positional references during the strain tracking process. Tensile tests were performed using a Test Resources Newton 100 series Tensile Tester with a 4,500-N load cell (Test Resources Inc., Shakopee, MN). Samples were preloaded to a load of 4.5 N at a positionally controlled rate of 1.27 mm/s. The preload was sustained for a half second before the major load was applied. The major load ramped up at a rate of 4.5 N/s to a total applied force of 22 N.

During tensile testing, the samples were filmed with a video camera to track the movement of the speckles (image shown in Fig. S3A). The load and position data from the tester were recorded using National Instruments data acquisition hardware (NI 9215) and LabVIEW software (National Instruments Corp., Austin, TX). Data were collected for the test over a window of 10 s at a sampling rate of 1,000 Hz.

The video recordings of the tensile tests were then processed to generate a strain map within the specimen using a custom MATLAB code [22]. Briefly, each video was segmented into a series of 21 images over a 10-s window of footage that captured the test, and a mesh was applied to the first image of the series using Abaqus software. To mesh the image, a 2-D planar part was created in Abaqus, which was modeled by tracing the image of the specimen, resulting in approximately 700 to 1,000 elements in the surface mesh. Once the mesh was applied, the data were exported to MATLAB, which converted the mesh intersections into individual nodes. The nodes were then scaled for the image using global coordinates from Abaqus and Gimp software. These nodes represented fixed points on the specimen’s surface, which could then be tracked across each frame based on their positions relative to the surrounding black paint speckles to calculate the approximate displacement and strain throughout the specimen. Figure S3B shows the nodes in red, overlaying the image of the strain tracking specimen. Based on the strain map, regions of consistent strain (in which there were no abrupt changes in the strain contour lines) were isolated to determine the Poisson’s ratio of the PVDF material using the average *x* and *y* strains in this region. An example of a strain map and the region of consistent strain used in the Poisson’s ratio calculations is shown in Fig. S4.

### Compression tests

Specimens for compression testing were taped with 2.5 cm × 2.5 cm squares of conductive copper tape to act as electrodes, applied to either side of the thin film. The area on the specimen covered by the copper squares was then cut out from the rest of the sample with scissors. Copper tape strips, 1.25 cm in width, were attached to the copper squares to serve as electrical leads, where alligator clips could connect the specimen to a modified Sawyer–Tower circuit (Fig. S5). Compression tests to determine the *d*_33_ piezoelectric coefficient of the PVDF were performed by dropping a 200-g cylindrical weight onto the PVDF specimen from a height of 2 cm. This method was used because sharp impact loads were found to produce more clearly discernable peaks in the voltage generated by the PVDF specimens, as opposed to gradually increasing loads on the tensile tester. The specimen was mounted on the lower grips of a tensile tester, with a load cell attached below the grips to measure the impact load of the 200-g weight. The weight was mounted in the upper grips of the tensile tester and was released during tests by loosening the grip, allowing the cylindrical weight to drop 2 cm onto the PVDF sample below. The electrical leads on the test specimen were connected to either side of a 100-nF capacitor, and the voltage generated by the specimen across the capacitor was measured and recorded in LabVIEW. This experimental setup is shown in Fig. S6. Five replicates of each test were taken for each sample.

The difference between the peak voltage and baseline voltage measured across the 100-nF capacitor in the modified Sawyer–Tower circuit, along with the impact load measured by the load cell, was used in Eq. 1 to determine the *d*_33_ coefficient of the specimen. The signal noise was smoothed using a 3-point Savitzky–Golay filter [23], and an example of a signal generated by this method is displayed in Fig. S7. This peak voltage is used to calculate the *d*_33_ coefficient of the sample:

$$d_{33}=\frac{Q}{F}=\frac{\mathrm{CV}}{F}$$ (1)

where *d*_33_ is the compressive piezoelectric coefficient, *Q* is the charge in the capacitor, *F* is the applied compressive load (1.96 N based on the 200-g weight), *C* is the capacitance of the capacitor (100 nF), and *V* is the voltage across the capacitor.

### FEA simulations

FEA simulations were performed using COMSOL Multiphysics software, specifically using the Piezoelectric Effect Multiphysics tool. A geometric model of a 2.54 cm × 2.54 cm film with a thickness of 70 μm was built. Domain partitions were created at an offset of 6.35 mm from either end of the film. The material applied to the model was the default PVDF from the COMSOL materials library. To replicate the material properties of the synthesized PVDF films, the elasticity matrix of the material was modified using the elastic moduli and Poisson’s ratios determined by the experimental data from the tensile testing and strain tracking. A free tetrahedral mesh was applied to the geometry with element sizes ranging from 0.46 to 2.54 mm. A floating potential was applied to the top face of the film, and the opposite face was grounded (shown in Fig. S8). Each of these faces was connected in series with a 100-nF capacitor in a simulated, modified Sawyer–Tower circuit.

A simulated compression test was performed to determine the *d*_33_ piezoelectric coefficient of the PVDF material. This was done by applying a constant, compressive load of 44.48 N to the top surface of the specimen, while applying a fixed constraint to the bottom surface (illustrated in Fig. S8). The *d*_33_ value of the simulated specimen was then calculated using Eq. 1.

### Statistical analysis

One-factor analysis of variance with post-hoc Tukey Honestly Significant Difference tests were performed to compare the means for elastic modulus, Poisson’s ratio, and both simulated and experimental *d*_33_.

## Data Availability

The data are available from the authors upon a reasonable request.

## Supplementary Materials

Supplementary 1 Fig. S1. Example stress–strain curves for PVDF specimens. Fig. S2. Evaporation rate of 2-butanone during PVDF synthesis. Fig. S3. A strain tracking specimen undergoing tensile testing (A), and a specimen with strain tracking nodes overlaying its image (B). Fig. S4. A strain contour map with a region of consistent strain noted with a rectangle. Fig. S5. Modified Sawyer–Tower circuit. Fig. S6. Experimental compression testing setup. Fig. S7. Example of a signal generated across a capacitor by a PVDF specimen during compression. Fig. S8. Simulation setup for compression testing.
