# Supplementary material for: Effects of Porosity on Piezoelectric Characteristics of Polyvinylidene Fluoride Films for Biomedical Applications
Source: BME Front. 2023 Jul 7;4:0009. doi: 10.34133/bmef.0009 (PMC10328389; doi:10.34133/bmef.0009)
Supplement: Supplementary 1 — Fig. S1. Example stress–strain curves for PVDF specimens. Fig. S2. Evaporation rate of 2-butanone during PVDF synthesis. Fig. S3. A strain tracking specimen undergoing tensile testing (A), and a specimen with strain tracking nodes overlaying its image (B). Fig. S4. A strain contour map with a region of consistent strain noted with a rectangle. Fig. S5. Modified Sawyer–Tower circuit. Fig. S6. Experimental compression testing setup. Fig. S7. Example of a signal generated across a capacitor by a PVDF specimen during compression. Fig. S8. Simulation setup for compression testing. [file bmef.0009.f1.docx]

**SUPPLEMENTARY MATERIALS**


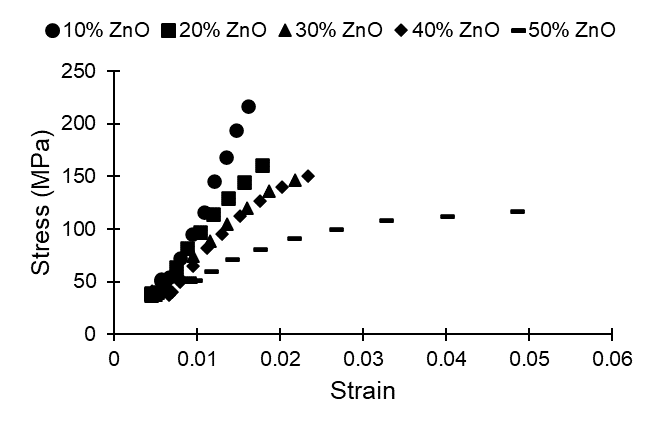


**Figure S1: Example stress-strain curves for PVDF specimens.** Overall, the elastic modulus and ultimate tensile strength of the specimens decreased with increasing wt% ZnO.


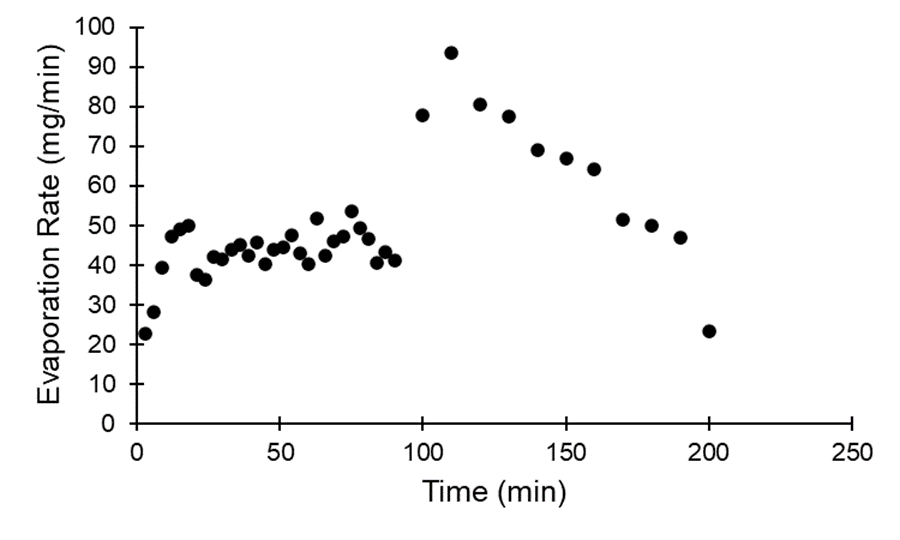


**Figure S2: Evaporation rate of 2-butanone during PVDF synthesis.**  The evaporation rate was a mean value measured from five pure PVDF samples fabricated. Samples were weighed at intervals of 3 minutes for the first 90 minutes of solution casting in an oven, and then in 10-minute intervals until samples were dry. The differences in weight were used to determine the evaporation rate of the 2-butanone.


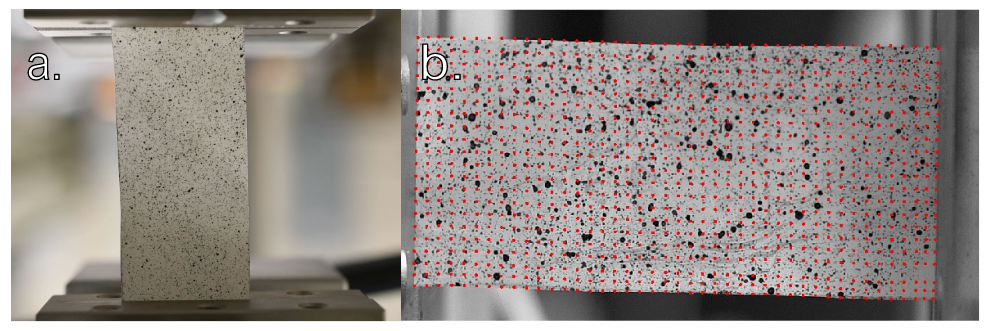


**Figure S3: A strain tracking specimen undergoing tensile testing (a), and a specimen with strain tracking nodes overlaying its image (b).**


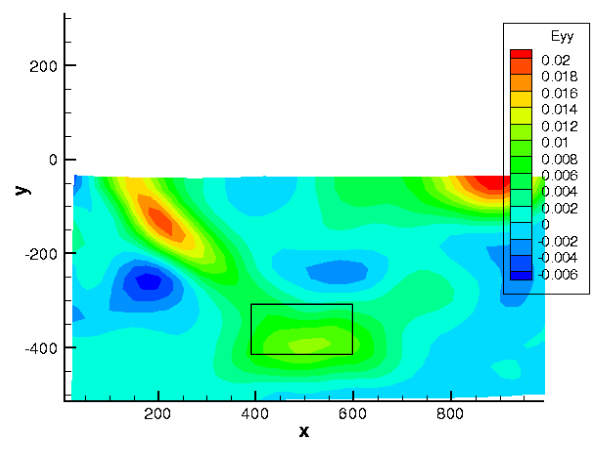


**Figure S4: A strain contour map with a region of consistent strain noted with a rectangle.** The rectangular area is a region of consistent strain (in which there were no abrupt changes in the strain contour lines) that was isolated to determine the Poisson’s ratio of the PVDF material using the average *x* and *y* strains in this region.

*
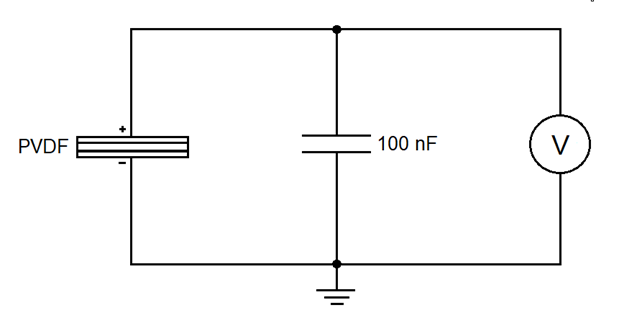
*

**Figure S5: Modified Sawyer-Tower circuit.** When a compressive impact load was applied to the PVDF specimen, the electric charges were produced on both sides of the specimen. The charges were calculated by the measured voltage across the capacitor times its capacitance (100 nF).


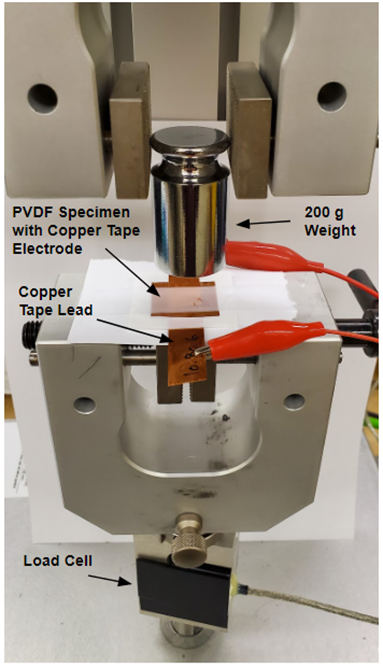


**Figure S6: Experimental compression testing setup.** The PVDF specimen was mounted on the lower grips of a tensile tester, with a load cell attached below the grips to measure the impact load of the 200 g weight that was mounted in the upper grips of the tensile tester. The weight was released during tests by loosening the grip to drop onto the specimen. The electrical leads on the test specimen were connected to either side of a 100 nF capacitor, and the voltage generated by the specimen across the capacitor was measured and recorded in LabVIEW.


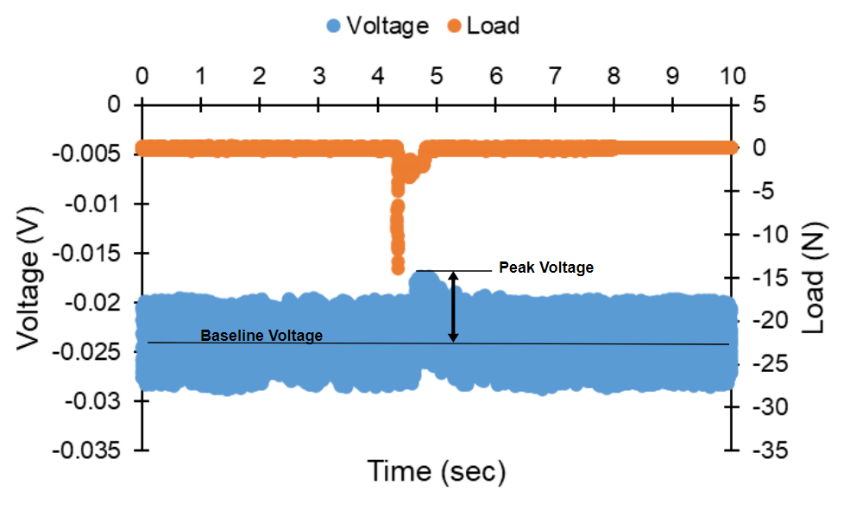


**Figure S7: Example of a signal generated across a capacitor by a PVDF specimen during compression.** The voltage was measured across the 100 nF capacitor in the modified Sawyer-Tower circuit and the impact load was measured by the load cell. The difference between the peak voltage and baseline voltage and the load were used in Equation 1 to determine the d_33_ coefficient of the specimen.


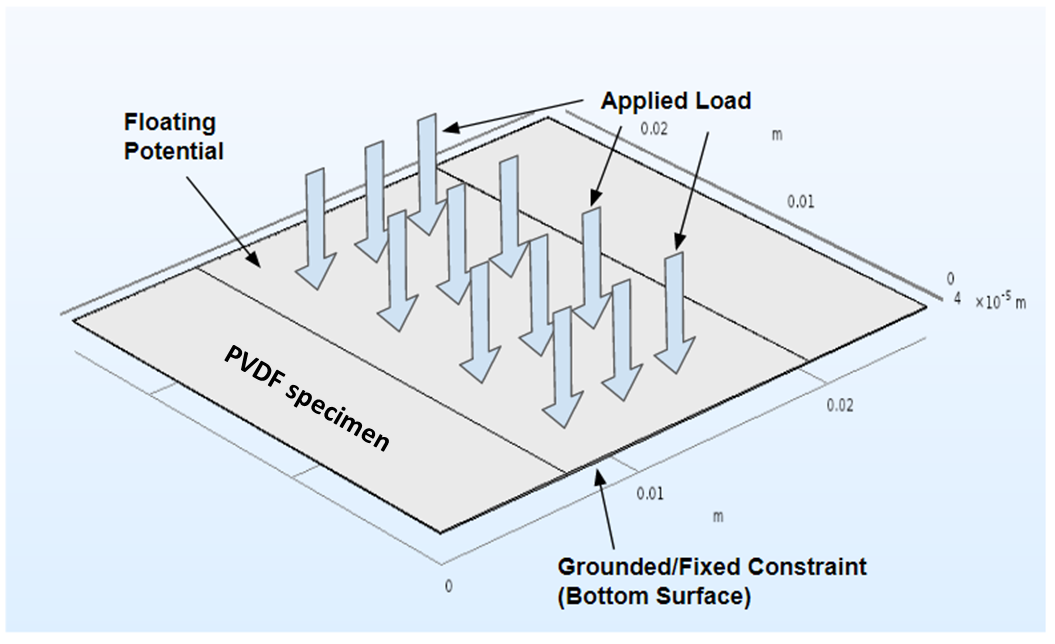


**Figure S8: Simulation setup for compression testing.** A floating potential was applied to the top face of the film, and the opposite face was grounded. A compressive load of 44.48 N was applied to the top surface of the specimen and a fixed constraint was applied to the bottom surface.
